# Ring-segment piezopolymer sensor optimized for cylindrical-wave detection in optical-resolution optoacoustic angiography with extended imaging depth

**DOI:** 10.1364/BOE.570072

**Published:** 2025-09-10

**Authors:** Alexey Kurnikov, Maxim Prudnikov, Daria Voitovich, Anna Glyavina, Anna Orlova, Marina Sirotkina, Wei Liu, Daniel Razansky, Pavel Subochev

**Affiliations:** 1 Laboratory of Ultrasound and Optoacoustic Diagnostics, Division of Radiophysics Methods in Medicine, Institute of Applied Physics, Russian Academy of Sciences, Nizhny Novgorod 603005, Russia; 2 Institute of Experimental Oncology and Biomedical Technologies, Privolzhsky Research Medical University, Nizhny Novgorod 603005, Russia; 3 Optical Imaging Laboratory, Harbin Institute of Technology, Shenzhen 518055, China; 4 Institute for Biomedical Engineering and Institute of Pharmacology and Toxicology, Faculty of Medicine, UZH Zurich, Rämistrasse 71, Zurich 8006, Switzerland; 5 Institute for Biomedical Engineering, Department of Information Technology and Electrical Engineering, ETH Zurich, Gloriastrasse 35, Zurich 8092, Switzerland; 6 pavel.subochev@gmail.com

## Abstract

Optical resolution optoacoustic (or photoacoustic) microscopy (OR-OAM) utilizing a 532 nm laser wavelength represents a promising approach for non-invasive visualization of superficial hemoglobin-rich structures. However, clinical translation of OR-OAM angiography typically faces a trade-off between achieving high contrast and resolution versus maintaining an extended depth-of-field at safe laser exposure levels. Gradient refractive index (GRIN) fiber lenses can provide an elongated optical focus preserved over a millimeter-scale length. However, developing coaxially aligned wideband acoustic detectors with high sensitivity remains challenging. Here, we introduce a piezopolymer PVDF-TrFE detector featuring a spherically-focused thin (100 μm) ring geometry with a 4.6 mm aperture and 1.5 mm working distance (NA = 0.84). Numerical modeling reveals not only an extended depth of field, but also an improvement in sensitivity compared to conventional full-aperture detectors. In vitro experiments using whole human blood demonstrated a 14 dB signal-to-noise ratio at a safe laser irradiance of 20 mJ/cm^2^. In vivo angiographic imaging of neonatal mouse cerebral vasculature and human cuticle confirmed the detector's capability to achieve a depth-of-field exceeding 1 mm, highlighting its potential for a broad range of biomedical applications.

## 1. Introduction

Functional and morphological analyses of the microvasculature are crucial in the early diagnosis of various vascular diseases, including Raynaud's syndrome, diabetes mellitus, venous and arterial insufficiency, and microvascular dysfunction [1–3]. Additionally, assessing the microvascular network provides valuable information about biological aging [4].

Among the standard methods of noninvasive visualization of the microvascular bed, videocapillaroscopy and laser Doppler methods [2], ultrasound examination (US) [5], computed tomography (CT) [6], magnetic resonance imaging (MRI) [7], and optical coherence tomography (OCT) [8–11] are most commonly used. However, these methods have a number of limitations, such as insufficient visualization depth, the need to use exogenous contrast agents or relatively low spatial resolution, which complicates a detailed assessment of the microvasculature.

To assess the state of the vascular network of biological tissues, the hybrid optoacoustic (or photoacoustic) angiography is increasingly being used [12–14]. This technology is based on the optoacoustic effect: when tissue is irradiated with short (nanosecond) laser pulses in the visible or near infrared range, broadband ultrasound waves are generated by the transient light absorption, which are recorded by ultrasound detectors.

Among the most common implementations of the method is optical resolution optoacoustic microscopy (OR-OAM or OR-PAM), in which tissues are probed with focused laser radiation, thereby providing high lateral spatial resolution, from several microns down to a sub-micron range [15]. The technique is commonly affected by hard compromises between optical depth of field and spatial resolution - as in other optical microscopy methods, tighter optical focusing limits the depth of field, leading to a loss of contrast outside the focal plane.

To obtain a three-dimensional image in systems with optically dense focusing, scanning along the vertical Z axis is often performed [16], which significantly slows down the imaging speed. Methods based on adaptive alignment of optoacoustic focusing have also been proposed to eliminate image distortions associated with the uneven surface of biological tissues [17]. In optical microscopy, non-diffracting beams (e.g., Bessel beam or Airy beam) are used to solve the problem of limited optical depth of field [18,19]. In optoacoustic microscopy, the depth of field has also been expanded by using Bessel beams [20–23]. Recently, a variant of optoacoustic microscopy with a needle light beam was proposed, which can expand the depth of field to 28 times the Rayleigh length using tunable diffractive optical elements [24].

An alternative way of extending the depth of field is to use gradient (GRIN) lenses with low numerical aperture, providing a depth of field greater than 100 μm and lateral resolution less than 10 μm [25,26]. In systems based on GRIN lenses, spherically focused piezoelectric detectors mounted on scanning XY stages are usually used to record signals. The lens itself is mounted in the center of the ultrasonic detector, ensuring a coaxial alignment of the optical and acoustic paths.

However, the problem of efficient recording of optoacoustic signals in OR-OAM systems with an extended optical focus remains. The narrow depth of field of spherical focused ultrasonic detectors limits the expansion of the depth of field in optoacoustic microscopy, making the extended Rayleigh optical length less effective. One of the previously considered solutions was to use a detector based on an acoustic Bessel lens, where the spherical concave surface is replaced by a conical concave surface to create an acoustic Bessel beam without diffraction, expanding the acoustic depth of field but sacrifices transducer sensitivity [27,28]. OR-OAM systems require high sensitivity of ultrasonic receivers [29,30], which is associated with the small size of the sources and, as a result, the rapid attenuation of the ultrasonic wave during propagation to the receiver [31]. To improve signal to noise ratio (SNR), spherically focused receivers with a hole through their center (ring-shaped detectors) are often employed for the excitation light delivery. This eliminates the need for complex optoacoustic combiners [32,33].

In this study, a new type of broadband detector based on a highly sensitive PVDF-TrFE piezopolymer film [34,35] is proposed for OR-OAM systems with an extended optical focus. Instead of traditionally used acoustically thick ring-shaped detectors, the new detector has geometry of an acoustic wavelength-matched (100 μm) ring segment with a high numerical aperture (NA = 0.84). This approach provides a significant expansion of the acoustic depth of field while simultaneously boosting sensitivity of the cylindrical wave detection.

## 2. Materials and methods

### 2.1. Numerical simulation

The OR-OAM system with an axially extended optical focus in the current study was implemented based on the GRIN lens from GRINTECH GmbH (Germany) with an aperture of 1 mm, a focal length of 8 mm and a numerical aperture of 0.06. Based on the GRIN lens characteristics, we used numerical simulation methods to determine the optimal geometry of the ultrasonic detector to increase the sensitivity of optoacoustic imaging. For this purpose, a numerical model was implemented in the Matlab environment (R2023a) based on two software packages: k-Wave for simulating the propagation of acoustic pulses [36] and the Monte Carlo method for simulating the distribution of illumination and absorbed energy [37].

Monte Carlo simulation was performed on a three-dimensional grid with a step of 1 μm. A two-layer medium was specified: water with biological tissues determined by the parameters of optical absorption μa, scattering μs and anisotropy g for a wavelength of 532 nm [38,39]. The vessels were specified as cylinders of different diameters with optical properties of hemoglobin for a wavelength of 532 nm [40]. The angular distribution of the photon trajectory was determined by the numerical aperture of the GRIN lens. The beam intensity distribution was Gaussian. Subsequent propagation of the acoustic wave from the source was performed using the k-Wave package on a two-dimensional grid with a spatial step of 5 μm and a time step of 1 ns. The medium was specified as homogeneous: water with a quadratic sound attenuation of 0.0022 dB/(MHz^2^×cm) [41]. The maximum supported frequency in the simulation was 150 MHz.

### 2.2. OR-OAM system based on GRIN lens and detector in the form of spherically focusing ring segment

**Fig. 1.**
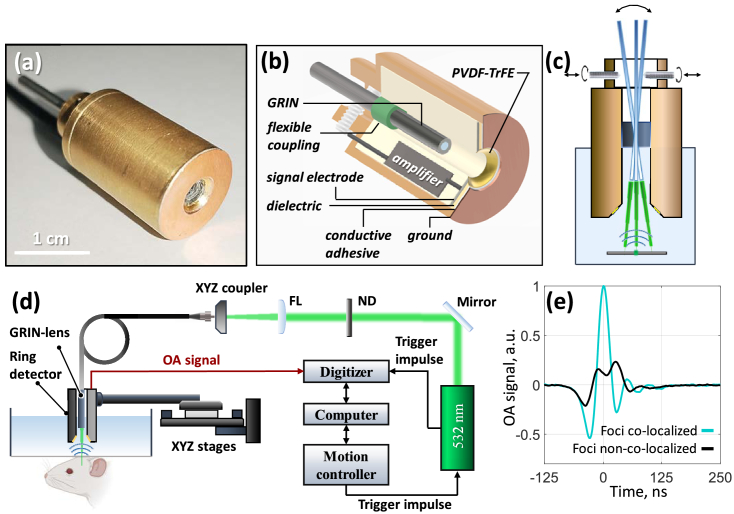
OR-OAM system based on GRIN lens and detector with spherically focusing ring segment geometry. (a) Photograph of ring-shaped detector based on PVDF-TrFE piezoelectric film. (b) Detector diagram containing its main parts. (c) Process of combining acoustic and optical foci. (d) OR-OAM system diagram (FL - focusing lens in the diagram, ND - neutral filter). (e) Example of signals recorded by the detector for combined and uncombined foci.

Based on the numerical simulation results, a detector with the geometry of a spherically focusing ring segment (annular detector) was developed based on a PVDF-TrFE piezoelectric film (Fig. 1(a)). The thickness of the piezoelectric film was 20 μm, which ensured a wide reception band (several tens of megahertz). The inner body of the detector was manufactured using an Elegoo Mars 4 DLP 3D printer (China). A 100 μm thick cupronickel sheet fixed to the plastic inner form of the detector was used as a signal electrode. At the first stage, a hole with a diameter of ∼ 4 mm was drilled in the metal sheet. Then the formed ring was ground to a size of 4.6 mm using a metal ball with a diameter of 5.5 mm. Thus, the signal electrode of the detector was a ring with an aperture of 4.6 mm focused into the center of a sphere with a radius of 2.75 mm. The factory metal coating was removed from one side of the piezoelectric film, after which the film sample was fixed to the prepared cupronickel electrode in the form of a focused ring using conductive glue. A broadband low-noise amplifier with a uniform gain band from 1 to 100 MHz and a gain factor of K = 30 was built into the inner casing. Then the detector was placed in an outer shielding casing made of brass, and the outer electrode of the piezoelectric film was connected along the periphery to the casing using a thin copper sheet, forming a common ground. Between the cupronickel sheet and the copper there was a dielectric layer 0.5 mm thick. A detailed diagram of the ring-shaped detector is shown in Fig. 1(b).

To align the optical focus angle relative to the acoustic focus within the detector’s central hole (designed for mounting the GRIN lens), a flexible coupling was implemented. The GRIN lens was rigidly secured inside a metal guide rod. One end of the rod was then fixed using the flexible coupling, while the other was held in place by three M2 clamping screws located on the rear side of the detector. The angular orientation of the metal rod and thus the attached GRIN lens was fine-tuned using these three M2 adjustment screws. Figure 1(c,e) shows the process of adjusting the optical focus and provides examples of optoacoustic signals for cases where the optical and acoustic focuses either coincide or do not coincide.

Figure 1(d) shows the general schematics of the OAM system based on the GRIN lens and the ring-shaped detector. An Onda 532 laser (BrightSolutions, Italy) was used as a laser radiation source. The radiation was coupled into a Nufern 460HP single-mode fiber (Coherent, USA) using a LA1540-YAG plano-convex lens (Thorlabs, USA) and an XYZ positioner (Oeabt CXYZ2-PM XYZ Translation Mount, China). A GRIN lens with an aperture of 1 mm and a focal length of 8 mm was soldered to the fiber end. The GRIN lens was fixed in the central hole of the ring-shaped detector. The ultrasonic detector was mounted on two horizontal linear piezoelectric stages V-408.132020 (PI micos, Germany) and a vertical stage (Standa, Lithuania). Optoacoustic signals were recorded using a 16-bit ADC (CSE25216, GaGe). The laser pulse triggering and signal recording were synchronized using trigger pulses from the motion controller.

### 2.3. Phantom and in vitro experiments

The system was initially validated through phantom and in vitro studies. The in vitro experiment consisted of recording optoacoustic signals while scanning a layer of whole blood. In the phantom experiment, a 7-μm-thick carbon microfiber was visualized, located at an angle to the scanning plane. The depth difference between the different edges of the carbon fiber was 2 mm with a horizontal distance of 5 mm. The scanning step of a 5 × 5 mm^2^ whole blood area was 100 μm, and the scanning step of the microfiber was 20 μm.

### 2.4. In vivo experiments

In vivo validation of the system was performed on a young female Balb/c mouse (3 weeks old, 12 g). Before the study, the animal was anesthetized with an intraperitoneal injection of a mixture of 40 mg/kg Zoletil (Valdepharm, France) and 10 mg/kg XylaVet (Alpha-Vet Veterinary Ltd., Hungary). The vascular network of the ear and brain (the scalp was removed, but the skull was preserved) of the mouse was visualized. After the study, the anesthetized animal was given a lethal 400 mg/kg doze of Zoletil (Valdepharm, France). All animal experiments were conducted in accordance with the European and Russian national guidelines for animal studies and were approved by the local ethical committee of the Privolzhsky Research Medical University.

One volunteer (a 27-year-old female) from the research team also participated in the in vivo experiment. Microvessels near the nail plate were visualized.

The imaging area of the mouse brain was 9 × 8 mm^2^, the mouse ear 8 × 8 mm^2^ and the microvessels near the human nail plate 7 × 7 mm^2^ with a scanning step of 20 μm. The imaging speed was approximately 3 minutes for an 8 × 8 mm^2^ area.

## 3. Results

### 3.1. Simulation results

**Fig. 2.**
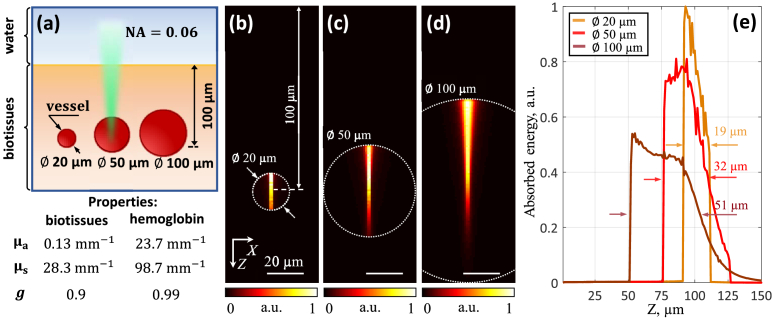
Absorbed energy in biotissues in the case of an axially extended optical focus of laser radiation. (a) Scheme of numerical simulation. (b-d) Distribution of absorbed energy in vessels of different diameters (20, 50, 100 μm) at a depth of 100 μm in biotissues. (e) Profiles of absorbed energy with FWHM.

Figure 2 shows the results of modeling the distribution of absorbed energy in vessels of different diameters located in biological tissues at a depth of 100 μm, when probing them with a beam having axially extended focus (NA = 0.06). For each vessel, the laser excitation beam was assumed to be focused in its center. Figure 2(e) shows the absorbed energy profiles along the Z axis, with the full width at half maximum (FWHM) being 19, 32 and 51 μm for vessels with diameters of 20, 50 and 100 μm.

The simulated vessel sizes sufficiently cover the spatial range from capillaries and postcapillary venules to arterioles. For the lens used in the GRIN system, the focal waist width in water can be theoretically estimated as *w_0_* ≈ 4.4 μm at a wavelength of 532 nm (*w_0_≈0.5λ_opt_/NA_opt_* [42], where *λ_opt_* is the optical wavelength and *NA_opt_* is the numerical aperture of the lens). In the case where the optical beam does not diverge much, as, for example, in Fig. 2(b,c) for vessels with a diameter of 20 and 50 μm, the shape of the absorbed energy can be approximated as a thin vertical cylinder (*w_0_*≪FWHM). That is, in the case of an axially extended optical focus, when the beam diameter is much smaller than the vessel diameter and the laser penetration depth into the vessel, the source of the optoacoustic wave will be an extended vertical cylinder.

**Fig. 3.**
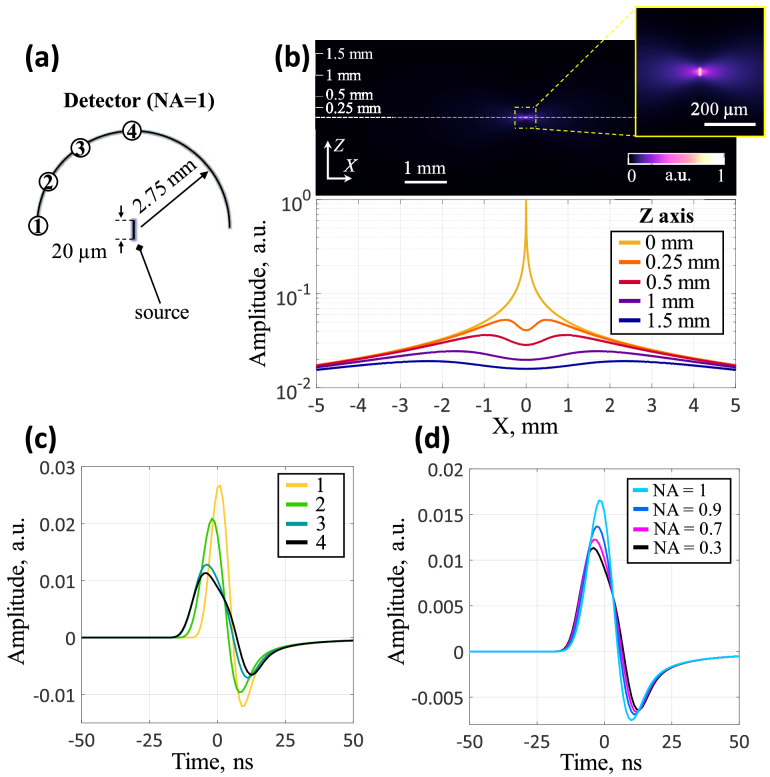
Detection of optoacoustic signals from an axially extended source. (a) Simulation scheme of signals from an axially extended source (numbers on the arc indicate signal recording points). (b) Spatial distribution of pressure from the source and pressure profiles along the X axis at different distances from the center (Z = 0) of the source. (c) Registered signals at different points of the detector (from the edge to the center). (d) Amplitudes of signals recorded by detectors with different numerical apertures.

To simulate the propagation of an acoustic wave, a vertical source with a thickness of 5 μm and a length of 20 μm was then placed in the center of the two-dimensional grid (Fig. 3(a)), since it was first necessary to estimate the sensitivity of the receiver to the smallest capillaries. Figure 3(b) shows the spatial distribution of pressure in the medium from an axially extended source, that has a dipole radiation pattern directed perpendicular to the main axis of the source. The pressure profiles along the X axis at different distances from the source (Z = 0 mm, 0.25 mm, 0.5 mm, 1 mm, and 1.5 mm) are also shown. The pressure profiles are characterized by a dip at X = 0 and the presence of maxima with increasing distance from the center.

Figure 3(c) demonstrates signals arriving at different points of the model detector with an angular coverage of 180° (NA = 1) and a focal length of 2.75 mm. Weaker low-frequency signals from the end of the thread arrive at the center of the detector (antennas #3 or #4 in Fig. 3(c)), and higher frequency signals with higher amplitude arrive at the edges of the detector (antennas #1 or #2 in Fig. 3c).

Figure 3(d) shows a comparison between the signals recorded by detectors with different acoustic numerical apertures (from 0.3 to 1). The results show that reduction of numerical aperture leads to a diminished signal amplitude. This is also easily explained by the result obtained in Fig. 3(c). Detectors with a low numerical aperture record only weak signals from the end of the source, while detectors with a higher numerical aperture capture signals with a higher amplitude from the edges.

**Fig. 4.**
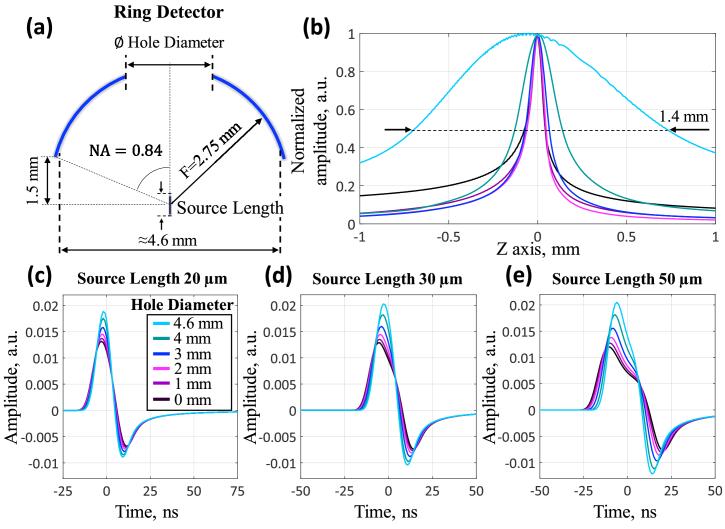
Optoacoustic signal detection using a spherically-focused ring detector. (a) Geometry of the detector and vertical source used in numerical modeling. (b) Acoustic reception field along the Z-axis for ring detectors with varying central aperture diameters (including thin-ring segment configuration), generated by a point spherical source. (c–e) OA wave detection efficiency versus central aperture diameter for vertically extended sources with 5-μm thickness and lengths of 20 μm, 30 μm, and 50 μm.

Figure 4 demonstrates the features of optoacoustic signal detection using a spherically-focused ring detector depending on central aperture diameter. The detector's working distance in simulations was set to Z = 1.5 mm to enable scanning and focal adjustment in real experiments (Fig. 4(a)). Part of this distance accommodates the detector housing. The 2.75 mm focal length was calculated based on the 1.5 mm working distance and 4.6 mm detector aperture, determined from the pressure profile maxima along the X-axis for a 20 μm axially-extended source (Fig. 3(b)).

Figure 4(b) shows acoustic reception field profiles along the Z-axis for ring detectors with different central aperture diameters when detecting signals from a point spherical source. As the central aperture diameter increases, the reception region along the Z-axis expands. At ≈4.6 mm diameter, when the detector becomes a thin ring segment, the depth-of-field substantially increases, creating a thread-like acoustic field in the axial direction.

**Fig. 5.**
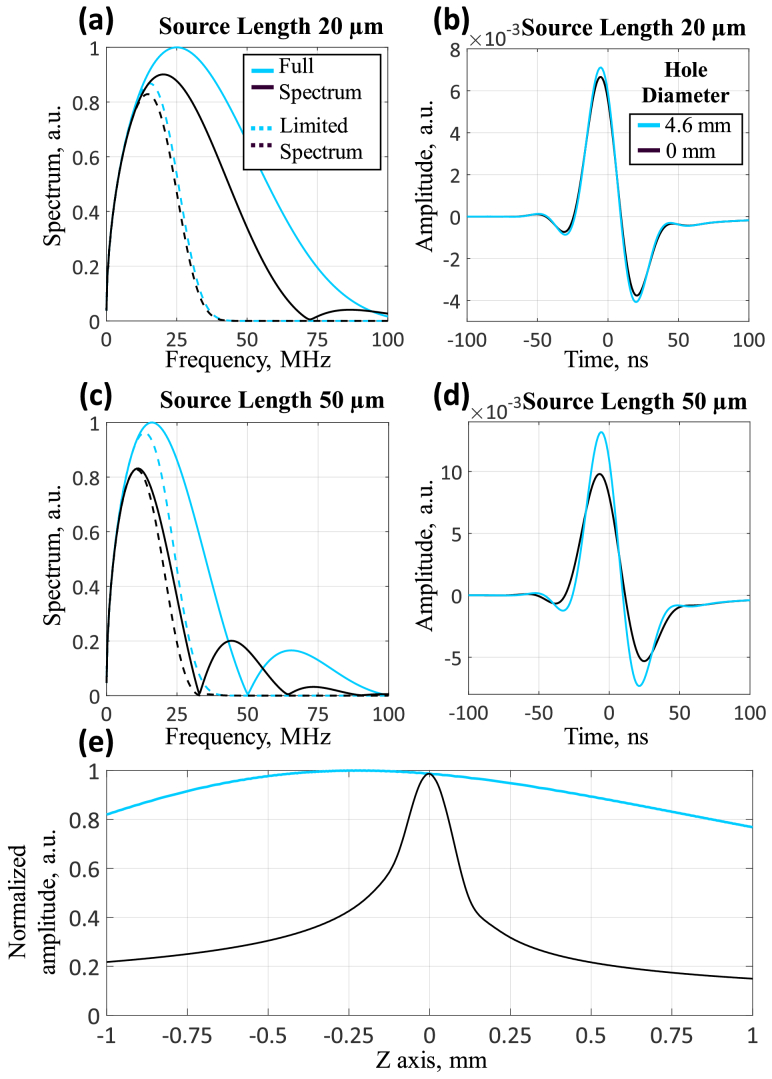
Effect of detector bandwidth limitations on reception efficiency in ring-shaped geometries. (a, с) Spectra of signals from vertical sources (20 and 50 μm) with full and limited (30 MHz) reception bandwidth. (b, d) Signals from vertical sources with a length of 20 and 50 µm in a limited reception band. (e) Acoustic reception field along the Z-axis in a limited frequency band.

Figures 4(c-e) present signal detection results for vertically extended sources (5 μm thick, 20/30/50 μm long, corresponding to depth-of-field for 20/50/100 μm vessels) using the ring detector with different central apertures. An increase in the central aperture leads to an increase in the signal, which follows from Fig. 3(c). This is especially pronounced with a greater source length.

Simulations revealed that the thin (100 μm) spherically-focused ring segment detector provides extended depth-of-field (1.4 mm) and enhanced sensitivity to axially extended sources at the focus. Signal amplitude increases by approximately 1.5 times for 20 μm sources and 1.7 times for 50 μm sources compared to a detector without central aperture.

This enhanced sensitivity of the thin-ring detector is achieved with an ultra-wide reception bandwidth (≈100 MHz), as evidenced by the simulated signal spectrum from a 20 µm axially-extended source (Fig. 5(a)). Modern piezoelectric materials can provide such bandwidth. However, with limited reception bandwidth (≈30 MHz, experimentally achieved), the sensitivity enhancement effect becomes less pronounced for the thin-ring segment: signal amplitude increases by ≈7% for 20 µm sources (Fig. 5(b)) and 30% for 50 μm sources (Fig. 5(d)) compared to a detector without central aperture. However, in this case, the acoustic field along the Z axis is further expanded (Fig. 5(e)).

Therefore, the thin spherically-focused ring-segment detector geometry not only significantly extends the depth-of-field - which is particularly valuable for optoacoustic systems with elongated optical focus - but also improves sensitivity to vertically extended sources, especially when using ultra-wideband detectors (≈100 MHz).

### 3.2. Results of phantom and in vitro experiments

**Fig. 6.**
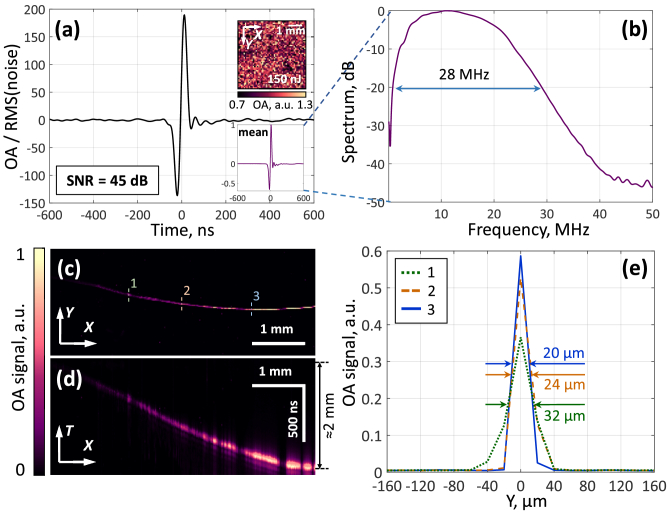
Phantom and in vitro tests of the OR-OAM system based on the GRIN lens and detector with spherically focusing ring segment geometry. (a) Typical optoacoustic signal recorded by the ring-shaped detector from whole blood (the insets show the projection of the maximum intensity for the blood layer and the average signal obtained from an area of 5 × 5 mm^2^). (b) Spectrum of the optoacoustic signal from whole blood. (c, d) Projections of optoacoustic images of 7 µm carbon fiber onto the XY and XT planes. (e) Lateral resolution of the system at different imaging depths.

Figure 6(a) shows a typical optoacoustic signal recorded by the ring-shaped detector during irradiation of whole blood with a focused laser pulse. The signal-to-noise ratio was 45 dB at a pulse energy of 150 nJ in the developed system. Considering the theoretical focal spot diameter of ∼5 μm (although in reality it can be much larger), the SNR from whole blood was 14 dB for a laser fluence of 20 mJ/cm^2^ (ANSI laser safety standard for a wavelength of 532 nm). The inset (Fig. 6(a)) shows the projection of the signal maxima recorded from the blood layer, demonstrating minor variations in amplitude. The observed spread can be due to both fluctuations in the laser radiation energy and possible inhomogeneity of the blood layer. Figure 6(b) shows the spectrum calculated from the averaged signal. The spectrum width was 28 MHz at the -20 dB level with a maximum at 11 MHz, which also emphasizes the broadband characteristic of the detector. Based on the spectral width *Δf* *=* *28 MHz*, the axial resolution of the detector can be estimated as 48 µm (*RA* *=* 0.88*с/*Δ*f).

Figure 6(c,d) shows the projections of the optoacoustic signals on the XY and XT planes obtained by scanning the microfiber. The results demonstrate a pronounced contrast from the fiber almost throughout the entire imaging depth (≈2 mm), which indicates a significant depth of field of the system provided by the combination of the GRIN lens and the ring-shaped detector.

Figure 6(e) shows the sections from Fig. 6(c), confirming the preservation of a high lateral resolution of no more than 30 μm. The observed local signal dips are explained by the use of a relatively large scanning step (20 μm) relative to the focal spot of the GRIN lens (≈5 μm). Nevertheless, the selected scanning step allows reliable registration of signals from submicron objects while maintaining a large field of view (>5 × 5 mm^2^).

The stronger signals from deeper segments of the carbon fiber compared to surface segments can be explained by a vertical misalignment of the optical focus relative to the acoustic one (the main priority for alignment of the focal points was by angle). The optical beam tends to broaden near the surface, which can lead to the generation of extended horizontal sources from a thin structure, such as a 7 µm carbon fiber, creating a blind zone in the immediate vicinity of the detector. Additionally, at small vertical distances from the detector, signal attenuation may occur due to partial refraction or reflection of acoustic waves.

The use of the time scale *T* instead of the spatial scale *Z* in Fig. 6(d) is due to the peculiarities of the detector ring geometry, where the transformation of the time delay *t* into the distance *z* requires the use of a nonlinear transformation, rather than a simple multiplication by the speed of sound (*ct*). The linear coordinate *z* can be calculated based on the radius of the ring (*r* = 2.3 mm) and the signal detection time at the receivers t as follows: *z* *=* *√(с^2^t^2^-r^2^)*, where с is the speed of sound in the medium (1.5 mm/μs in water).

### 3.3. Results of in vivo experiments

**Fig. 7.**
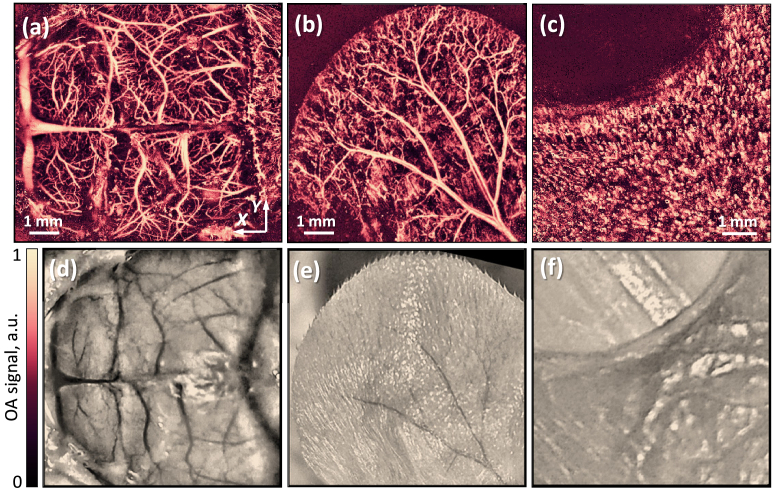
In vivo imaging results for the developed system. (a, b, c) Optoacoustic images of the brain vasculature (without scalp), the ear of a young mouse, and microvessels near a human nail plate. (d, e, f) Photographs corresponding to the optoacoustic images.

Figure 7 shows the in vivo imaging capabilities of the system. The lower panels show photographs of the brain of a young (3 weeks) scalped mouse, a mouse ear, and tissue near a human nail plate. The upper panels of the figure show the resulting optoacoustic images, which clearly distinguish both large vessels with a diameter of up to several hundred microns and tiny capillaries.

The energy of laser pulses in the in vivo experiments was less than 700 nJ. Low-pass filtering with a cutoff frequency of 30 MHz was applied to the recorded signals to suppress high-frequency noise. The cutoff frequency was determined based on the spectrum obtained in an in vitro experiment with whole blood. Then the Hilbert transform was applied to the signals to move them into the positive region of the signals. After that, projections of the signal maxima were constructed on the XY plane. The contrast between the smallest and largest vessels in the image was equalized using contrast-limited adaptive histogram equalization (CLAHE).

**Fig. 8.**
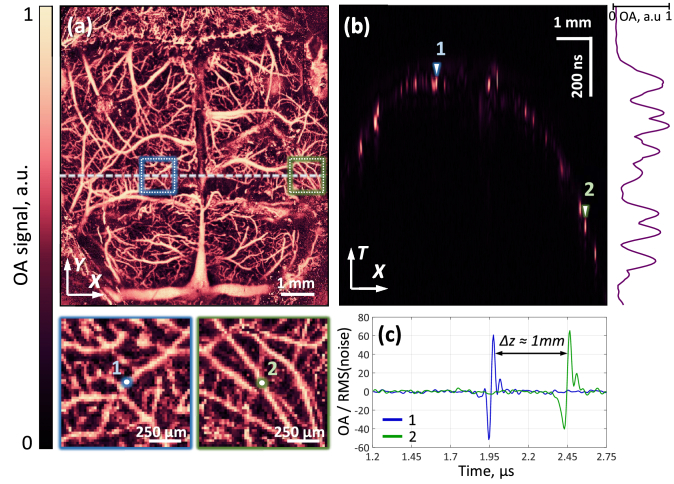
Achieved depth of field in in vivo experiments. (a) Projection of the OA image of the mouse brain vasculature onto the XY plane with highlighted areas (blue and green dotted squares) of microvessels located at different depths. (b) B-scan containing vessels 1 and 2 from the highlighted areas. (c) Optoacoustic signals from vessels 1 and 2.

Based on the results of visualization of the mouse brain vasculature, a detailed analysis of the depth of field achieved in vivo has been performed (Fig. 8). A separate B-scan (Fig. 8(b)), containing predominantly small vessels, shows a fairly uniform distribution of signals with depth, which is also confirmed by the profile of the maxima to the right of Fig. 8(b). In the insets of Fig. 8(a), the dotted squares mark the areas with microvessels at different visualization depths, demonstrating comparable image clarity. As can be seen from Fig. 8(c), the optoacoustic signals from vessels 1 and 2 (selected from the designated areas in Fig. 8(a)) are close in amplitude, despite the difference in their depth (∼1 mm), which also demonstrates the extended depth of field of the system during in vivo visualization.

**Fig. 9.**
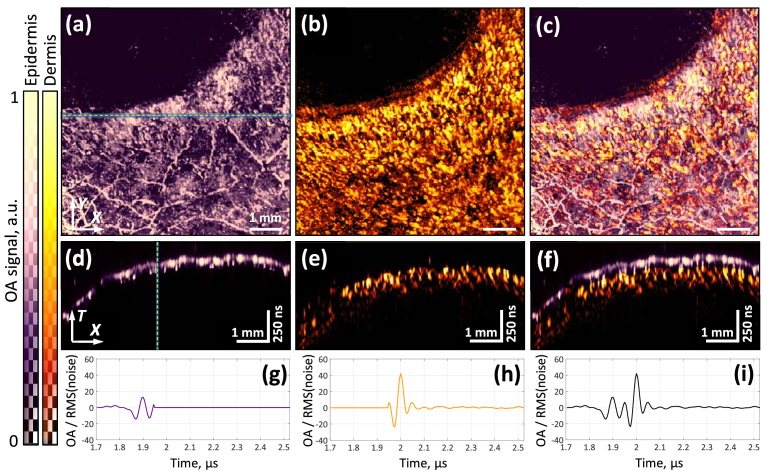
Optoacoustic visualization of the microvasculature near the human nail plate. (a, b, c) Projections onto the XY plane of optoacoustic images of separated layers of the epidermis, dermis, and their combined image. (d, e, f) B-scans corresponding to the dotted line in (Fig. a). (g, h, i) A-scans corresponding to the dotted line in (Fig. d).

Figure 9 shows the detailed results of visualization of human tissues near the nail plate. The original three-dimensional volume contained two layers, which could be clearly separated during optoacoustic visualization with the developed system. Figure 9(a,b) shows the projections of optoacoustic signals on the XY plane for separate layers: the epidermis and part of the dermis. The epidermis was characterized by the presence of various inhomogeneities, e.g. extended thin structures, while the dermis contained many tiny capillaries, orderly oriented towards the nail plate. Figure 9(c) shows a combined image of both layers. Figure 9(d-e) shows the B-scans (the selected area corresponds to the dotted line in Fig. 9(a)), which clearly demonstrate the spatial position of the layers relative to each other. Separation of the layers can also be observed in individual A-scans (Fig. 9(g-i)).

Visualization of individual layers of biological tissues and their combined image was performed using Avizo Software 2024.1 (Thermo Fisher Scientific), where each layer had its own translucent color scale.

## 4. Discussions

The developed optoacoustic microscopy system based on a GRIN lens and a PVDF-TrFE detector with spherically-focused ring-segment geometry demonstrates significant potential for both fundamental biological research and diagnosis of vascular diseases at the microcirculation level. Its key advantages include extended depth of focus combined with high spatial resolution and SNR.

The system is particularly valuable for assessing microcirculatory networks in superficial tissues in diseases such as diabetes, Raynaud's syndrome, venous and arterial insufficiency. Changes in the microvascular network may indicate underlying cardiovascular and metabolic pathologies [4]. Skin serves as a convenient target for non-invasive diagnosis of systemic vascular disorders due to the close correlation between cutaneous microcirculatory changes and pathological processes in other organs and tissues [43].

Recent research has increasingly focused on translating optoacoustic systems into clinical practice [44]. This requires high-speed, compact and portable OA devices [45,46]. Optoacoustic systems are often integrated with ultrasound systems, facilitating their clinical adoption [47].

The GRIN lens-based optoacoustic imaging system with ring-segment detector currently faces limitations such as imaging speed (compared to optical beam scanning systems) and prolonged signal integration due to the low signal detection efficiency with thin ring-segment detectors. Yet, the system can be implemented as portable medical cart with integrated robotic arm, as seen in commercial clinical systems [48,49]. Such solution may become a powerful tool in oncology, e.g. for studying vascular patterns in benign and malignant skin tumors to improve differential diagnosis, developing new prognostic factors or determining disease stage [50]. Other potential applications include regenerative medicine for investigating wound healing [51] as well as endocrinology for assessing limb perfusion in diabetic patients and monitoring diabetic ulcer regeneration during treatment [52].

## 5. Conclusions

We developed a PVDF-TrFE piezopolymer detector with a unique spherically focusing ring segment geometry (4.6 mm aperture, 1.5 mm working distance) for GRIN-lens-based OR-OAM systems. Numerical modeling demonstrated the superior performance of our design in systems with extended optical focus, showing over 50% sensitivity improvement compared to conventional full aperture focusing detectors (subject to wide reception bandwidth ≈100 MHz). Experimental validation confirmed the detector's high efficacy, achieving clear whole blood signals with 14 dB signal-to-noise ratio at safe laser fluence levels (20 mJ/cm^2^). In vivo studies revealed the system's capability for detailed microvascular imaging, including mouse cerebral vasculature and human cuticle capillaries, while maintaining high spatial resolution (20-30 μm) and SNR across an extended depth-of-field (>1 mm).

## Data Availability

Data underlying the results presented in this paper are not publicly available at this time but may be obtained from the authors upon reasonable request.
